# *Notes from the Field:* Crimean-Congo Hemorrhagic Fever Outbreak — Central Uganda, August–September 2017

**DOI:** 10.15585/mmwr.mm6722a6

**Published:** 2018-06-08

**Authors:** Susan Kizito, Paul E. Okello, Benon Kwesiga, Luke Nyakarahuka, Stephen Balinandi, Sophia Mulei, Jackson Kyondo, Alex Tumusiime, Julius Lutwama, Alex R. Ario, Joseph Ojwang, Bao-Ping Zhu

**Affiliations:** ^1^Uganda Public Health Fellowship Program, Kampala, Uganda; ^2^Uganda Virus Research Institute, Entebbe, Uganda; ^3^Ministry of Health Kampala, Uganda; ^4^CDC, Uganda; ^5^Division of Global Health Protection, Center for Global Health, CDC.

On August 20, 2017, physicians in two noncontiguous districts in central Uganda (Kyankwanzi and Nakaseke) reported two unrelated cases of Crimean-Congo hemorrhagic fever (CCHF). CCHF is the most widespread tickborne viral hemorrhagic fever in the world and represents a global health security threat (*1*–*3*); a single case of CCHF constitutes an outbreak. Humans are infected through tick bites or contact with the blood or body fluids of infected persons or animals. Treatment of infected patients is supportive, and the case-fatality rate ranges from 3%–40% (*2*,*3*). No licensed vaccine is available (*2*). Although CCHF cases were first reported in Uganda between 1958 and 1977, no subsequent cases were reported until 2013, when enhanced viral hemorrhagic fever surveillance capacity began to identify CCHF outbreaks (*3*–*5*).

The two cases were confirmed by serology and reverse-transcription–polymerase chain reaction (RT-PCR) testing at the Uganda Virus Research Institute (UVRI), a specimen referral system established in 2013 with assistance from CDC/Uganda in an effort to advance the global health security agenda (*5*). Upon confirmation of the two cases, the Uganda Ministry of Health deployed a team to investigate on August 22, 2017. A suspected case was defined as sudden onset of fever >100.4°F (38°C) for ≥3 days during July 1–September 30, 2017, plus either spontaneous bleeding or bruising, or laboratory evidence of unexplained leukopenia or thrombocytopenia in a resident of either of the two affected districts. A confirmed case was one that tested positive for CCHF by both RT-PCR and immunoglobulin M serology (*4*).

To identify cases, medical records of patients seen at area referral hospitals with fever and bleeding symptoms were reviewed. An active case search was also conducted in the affected communities. In addition to the two initial patients with confirmed cases, both of whom survived, among 23 medical records reviewed, five additional patients met the suspected case definition, two of whom died. Symptom onset occurred during July 9–September 17, 2017. Specimens were unavailable for confirmatory CCHF testing from the five patients with suspected cases. All cases occurred in men aged 19–87 years; no secondary cases were found.

A case-control study was conducted to compare potential exposures of case-patients and controls. Controls (four per case) were selected from among case-patients’ asymptomatic neighbors, matched by sex and age. Data on potential exposures, including tick bites or barehanded crushing of ticks, milking or butchering livestock, butchering wildlife, and caring for sick persons, were collected using a standardized questionnaire. Because infected animals might develop high viral load titers yet remain asymptomatic (*6*), blood samples were collected from cattle and goats from two farms where patients with confirmed cases worked and were tested using an enzyme-linked immunosorbent serologic assay.

Tick exposure was reported by four of seven suspected and confirmed case-patients and three of 28 (11%) controls (Mantel-Haenszel odds ratio = 11.0; Fisher exact 95% confidence interval [CI] = 1.1–112.0). At farms where patients with confirmed cases worked, 37 (60%) of 62 cattle and 5 (24%) of 21 goats were found to be seropositive for CCHF. Animals from these farms were quarantined for 1 month, during which time farm owners and workers were advised to use adequate protection when handling them.

A district rapid response team in each of the two affected districts was activated on August 23, 2017, including establishment of an emergency hotline for case reporting. Area hospitals designated isolation units for screening and isolating patients with suspected cases and collecting blood samples for testing at UVRI. Health care workers were trained in patient management and infection control; and district veterinary officers reached out to farmers, especially those whose farms had seropositive animals, regarding tick control (e.g., dipping livestock in acaricide concentrates). Community outreach concerning the signs, symptoms, and complications of CCHF and preventive measures was conducted via radio during August 24–September 30, 2017. Area residents were advised to avoid handling ticks with bare hands and to wear protective gear such as gloves, boots, and clothes to minimize their exposure risk while grazing livestock. No subsequent cases were reported after these measures were implemented. The rapid and coordinated response to this outbreak demonstrated the significant progress made to enhance global health security in Uganda.
